# An Examination into the Causes of the Declining Reputation of the Medical Faculty of the University of Edinburgh, &c.

**Published:** 1834-04-01

**Authors:** 


*An Examination into the Causes of the declining Reputation of the Medical Faculty of the University of Edinburgh; and the Origin of another Class of Medical Professors, commonly called "Private Lecturers," &c.*—Edinburgh, 1834. 8vo. pp. 59.

The author of this pamphlet is very angry with matters as they are, and still more angry with matters as they are about to be. He is terribly afraid that the new curriculum devised by the Edinburgh people will have the effect of preventing men with slender purses from entering the medical profession, and then goes on to say,

"Who will patiently attend a sick person night and day, encountering boisterous weather, cold and fatigue, with the greatest devotion?—the rich man, or he who has nothing to depend upon but a good character and his own exertions? Who will condescend most readily to perform a thousand little acts of kindness and attention? Who will call on an invalid twice or thrice a day, and bear with numberless whimsical rebukes, and be wearied out with long stories of perhaps imaginary evils? Who will take most trouble in soothing the mind of the afflicted, or comforting the broken-hearted?—the wealthy man, or he who has nothing but his own exertions to depend upon?

"If lords and ladies only were subject to pain and disease, it might be well to limit the profession to a few physicians, polished up to the highest pitch of refinement in all the arts and sciences. But when we look around us, and see the diseases and wants of multitudes in the various grades of society requiring the assistance of thousands of medical men, we may well ask, if such an extended and therefore very expensive medical education be persevered in, who will attend the middle and lower classes, not to speak of the poor? The metropolis and large towns may be well supplied, but who will go to practise in the smaller towns or country districts?" (P. 25.)

A sad prospect indeed for the country towns, and for invalids who love to take a physician, like a saline draught, *ter in die!* However, let not the sick despair; for, though the doctors *in posse* may be unbearably fastidious, and shockingly over-educated, many of those *in esse* meet their patients on more equal terms; at least, if we may believe what follows.

"Every one connected with medical education in Edinburgh (except, perhaps, the professors themselves,) is aware that by far too large a proportion of candidates obtain degrees in physic, and that many persons have passed the ordeal who were notoriously inattentive to their studies, and dissipated in their habits, till perhaps within a few months of the day of examination: others, very industrious, but so stupid that they never could preserve a respectable status among their fellow-students, have likewise passed, to the no small surprise of their companions. So frequently have such circumstances occurred, that it is no uncommon matter to hear of Edinburgh graduates having made a bad appearance, nay having been rejected, not only at the College of Physicians in London, and College of Surgeons, but before the Worshipful Company of Apothecaries, at Apothecaries' Hall. The writer has known many who have received their degrees in Edinburgh, who were not to be trusted with the life of a cat, and who could not write a decent letter in the mother tongue, either in point of style or orthography, although they wrote, or rather got the credit of writing, a dissertation in Latin! Indeed, it is stated by persons connected with the Medical Corporations in London, that they possess letters from persons with Edinburgh diplomas that are quite disgraceful." (P. 41.)

For ourselves, we do not expect the medical world to be turned upside down by the curriculum in question. We are quite sure that there will always be doctors of all marketable prices and qualities; some, like the refined physicians whom our author dreads, who would interrupt a gouty alderman in his favourite story, or contradict a maiden aunt, as if unconscious of her India bonds; and others who will "bear with numberless whimsical rebukes" on the lowest terms, and cheerfully submit to "be wearied out with long stories of perhaps imaginary evils."

There is one point on which the author is clearly in the right. The town-council ought to have nothing to do with electing professors; they are obviously disqualified, both by ignorance and long habits of jobbing, to judge of professional merit. The following table is strange, if true, as they say in America:

" *Comparative Statement of the Number of Students attending some of the Elementary Classes in the Medical Schools in Edinburgh, during the last two Sessions; distinguishing the University from the Extra-Collegiate Lecturers.*

| CLASSES. | University. | | Extra-Collegiate Lecturers. | | REMARKS. |
|---|---|---|---|---|---|
| | 1832-3 | 1833-4 | 1832-3 | 1833-4 | |
| Anatomy ........ | 186 | 167 | 460 | 470 | N.B. It is difficult to obtain an accurate list of all students attending extra-collegiate classes. The Album of the College of Surgeons cannot give so correct a view as that of the University, as perhaps one fourth part of the students, not intending to take their diplomas in Edinburgh, do not enrol themselves. But the number stated, the writer thinks, is under rather than above the mark. |
| Chemistry ...... | 360 | 232 | 65 | 170 | |
| Surgery ........ | 123 | 202 | 376 | 296 | |
| Practice of Physic | 136 | 128 | 210 | 147 | |
| Practical Anatomy | 108 | 109 | 450 | 440 | |
| Practical Chemistry | 111 | 8 | 100 | 200 | |
| Midwifery ...... | 150 | 122 | 190 | 180 | |
| Clinical Surgery .. | 99 | 77 | no class | 75 | |

